# Editorial: The influence of flourishing and its associated factors on the mental health and well-being of individuals

**DOI:** 10.3389/fpsyt.2025.1734470

**Published:** 2025-11-06

**Authors:** Giancarlo Lucchetti, Barbara Badanta, Rocío de Diego-Cordero, Homero Vallada, Juliane Piasseschi de Bernardin Gonçalves

**Affiliations:** 1 Department of Medicine, School of Medicine, Federal University of Juiz de Fora, Juiz de Fora, Brazil; 2 Department of Nursing, Schools of Nursing, Physiotherapy and Podiatry, University of Seville, Seville, Spain; 3 Departamento de Psiquiatria, Faculdade de Medicina da Universidade de São Paulo (ProSer), São Paulo, Brazil; 4 Department of Research, Instituto HPV (IHPV), São Paulo, Brazil

**Keywords:** flourishing, mental health, virtues and character, psychiatry, well-being

The mental health burden is increasing, posing challenges to healthcare systems worldwide (1). Aiming to reduce this burden, new models of care are using some concepts of positive psychology to generate observational evidence and to propose interventions, such as values, virtues, and flourishing. Flourishing is defined by VanderWeele as “the relative attainment of a state in which all aspects of a person’s life are good including the contexts in which that person lives”, which includes domains of human life such as (i) happiness and life satisfaction; (ii) health, both mental and physical; (iii) meaning and purpose; (iv) character and virtue; and (v) close social relationships and four pathways, i.e., family, work, education, and religious community (2). These domains have been increasingly studied recently and are based on a less disease-oriented and a more person-centered view of healthcare.

Although there is a consolidated literature investigating the factors associated with mental health and well-being among different populations, more evidence is needed concerning the role of values, virtues, and flourishing. This Research Topic provides a deeper understanding of the role of flourishing and its associated factors on the well-being and mental health of different populations, bringing new insights and novel approaches to address individuals’ mental health.

A total of 13 articles were accepted, embracing reviews, observational studies, and intervention studies on flourishing, positive emotions, virtues and values, social support and coping and religiousness. In this topic, prediction models were developed for life satisfaction (Nguyen et al.), the relationship between sense of humor and positive mental health (Piñar-Rodríguez et al.), and the mediating role of positive emotion were assessed (Wang et al.). Social support was evaluated through social mobility (Zhang et al.), and coping strategies were assessed during the COVID-19 pandemic (Low et al.). Virtues and values such as altruism and forgiveness were found to be important aspects for individuals (Bonete et al.; Yuan and Zhao), and religiousness was associated with quality of life (Bonilla Sierra et al.). The more general concept of flourishing was assessed among health professional students (Shdaifat et al.), the impact of life situations on flourishing was assessed in adults (Brydges et al.), and the mechanisms for the relationship between flourishing and health were reviewed (Bastos et al.). Interventions also had a place in this topic, including the protocol for a self-directed RECEIVE Forgiveness workbook (Cowden et al.) and a quasi-experimental study investigating a Flourishing intervention on individuals with depressive symptoms (Braghetta et al.).

The evidence obtained from this Research Topic improves our knowledge in this field, shifting our view of health towards a more holistic and integrative care and opening discussion to the role of virtues, values and positive emotions as potential markers of well-being. The variety of themes and articles supports the potential of research dealing with flourishing and may shed light on future directions of research. Below, a brief discussion of the strengths and weaknesses of this field will be provided, aiming to serve as a summary and a perspective for those entering in the Flourishing research.

The old definition of health, i.e. “a state of complete physical, mental and social and well-being and not merely the absence of disease or infirmity” (3) is changing significantly. In 1979, the term “salutogenesis” was used aiming to replace the binary distinction of disease and absence of disease into a continuum model (4), which supports a more realistic and humanitarian notion of health.

The term flourishing, used as a comprehensive well-being term, derives from the notion of eudaimonia by the philosopher Aristotle. To Aristotle, eudaimonia requires an active development of values, such as good, courage, virtues, justice and intellect, composing an ethical behavior (5). Evolving from this Aristotelian concept, flourishing was conceptualized as a positive functioning and relation to others, autonomy, personal growth, and purpose in life (6). Adding to the relationships and meaning, other crucial elements to flourishing were presented, such as positive emotions, engagement, and accomplishment (7).

Drawing attention in the health field, flourishing was also described as the positive and good pole of mental health, opposed to languishing (8). Considered as a presence of mental health and absence of mental disorders, flourishing overlapped with mental health as a concept from the early 2000s onwards. Described as high levels of psychological and subjective well-being (9), flourishing was also associated with positive emotion and feelings and high functioning levels (10).

More recently, the human flourishing concept expanded to a broader vision, including its relationship with the environment as an important condition to flourish (2). This perspective connects more closely the currently flourishing concept to Aristotle’s eudaimonia concept, since it integrates the social environmental and political aspects into the equation (11). In this sense, clearly, the complexity of this concept also cuts across different cultures.

A recent initiative to map flourishing determinants across several cultures and countries was launched in 2022 (12). Initial data have shown interesting findings, pointing to the fact that different countries show different levels of flourishing, which relied upon sociodemographic, social and psychological condition. Nevertheless, some developing countries surprisingly presented greater levels of flourishing as compared to richer and developed countries. Additionally, flourishing domains have been associated with different outcomes, such as lower mortality risk, and greater purpose in life (13) and altruistic behavior (14). Evidence supports then that flourished individuals seem to function better in different aspects of life (15).

The possible mechanisms for these findings are not totally understood, and multifactorial explanations are the best fit to explain the possible benefits of this flourishing in health. Different studies have shown that higher levels of flourishing domains may enhance positive emotions and thoughts, strengthen healthier behaviors and promote a positive cycle of well-being. Positive spiral lifestyle proposes that once an individual experiences a healthier behavior, stronger motivation will create, unconsciously, a way to maintain that behavior (16). These effective changes can support intrinsic development, which can sustain new, healthier behavior (17).

Despite all this promising evidence and the ongoing discussion concerning flourishing, there are limitations related to the measurement of this concept. Although scales to assess flourishing exist, most of them focus on hedonic and eudemonic aspects (18). The lack of consistency among instruments makes it difficult to adequately address flourishing dimensions, potentially biasing empirical research (18). Besides, there is also lack of a more consensual definition of flourishing itself. Since it is a multidimensional concept with roots in the Aristotelian idea of eudaimonia, several contemporary interpretations have taken ownership of the term flourishing (11), describing it with different well-being flavors. It is important for empirical advances in this field that researchers choose the referential framework of flourishing they want to pursue.

Moreover, interventions approaching virtues and values have been proposed in the last decades, showing improvements in mental health and well-being (19, 20). The type of intervention can vary, considering group or individual, and the format can focus on one specific thematic or multi-component virtues, also varying the duration of sessions (19). However, there is a lack of research that builds the intervention based on a more comprehensive framework of flourishing. Including this multi-component flourishing interventions may promote “chain” effect to improve health, by targeting a holistic approach to well-being, as seen in the favorable results obtained in one of the papers published in our Research Topic (Braghetta et al.).

Some future perspectives arise from our Research Topic, which include the necessity of strengthening the philosophical aspects of the concept and the development of instruments that rely on comprehensive and muti-dimensional frameworks that can enhance the quality of the scales currently available. Finally, new studies should investigate flourishing interventions that address several virtues and values, instead of relying on single domains, providing tools to individuals to maintain log-term flourishing.

In conclusion, our Research Topic corroborates with the ongoing research on flourishing, adding evidence to this field, but also highlighting possible limitations and proposing future directions for research. Researchers and health professionals should be aware of this comprehensive view of well-being to improve their healthcare and shift to a more person-centered aspect of health.
